# Urological Implications Associated with the Use of Recreational Drugs: A Narrative Review

**DOI:** 10.5152/tud.2022.22066

**Published:** 2022-07-01

**Authors:** Mudassir Wani, Musaab Hamdoon, Greg Dewar, Philippa Buakuma, Sanjeev Madaan

**Affiliations:** 1 Urology Trainee, Royal Glamorgan Hospital, Cardiff, UK; 2 Urology registrar, Royal Liverpool and Broadgreen university hospital, UK; 3 Registrar Dunedin Hospital, Southern District Health Board, New Zealand; 4 General Practitioner, Gravesend, Kent, UK; 5 Consultant Urological Surgeon, Darrent Valley Hospital, UK & Visiting Professor, Canterbury Christchurch University, UK

**Keywords:** Illicit drugs, lower urinary tract symptoms, ureter, kidney, infertility

## Abstract

About 275 million people worldwide aged between 15 and 64 years used drugs at least once since 2016. Initial estimations suggest that 13.8 million young people between 15 and 16 years used cannabis every year. Recreational drug use contributes significantly to mortality as well as physical and mental health problems. A number of urological complications can arise from the use of common and emerging recreational drugs which can present as wide spectrum affecting lower and upper urinary tracts, kidneys, sexual organs as well as sexual dysfunction. In order to effectively manage these issues, urologists need to be cognizant of these complications in their patients, particularly among youths. This review attempted to consolidate available data and provide insight into this issue; however, further population-based epidemiological studies are needed to provide necessary guidelines.

**Main Points**

- Illicit drug abuse can have an adverse impact on the urological system.
- Urological complications can present with unknown and unexpected clinical manifestations.
- Urologists must be aware of atypical presentations of genitourinary complications associated with illicit drug abuse.

## Introduction

Illicit or recreational drug use is a worldwide problem affecting large populations but is commonest among young adults. According to the World Health Organization, 450 000 people died last year due to drug use.^1^ Of these, 167 750 were associated with drug overdoses. Opioids continue to cause the most harm and are associated with 76% of illicit drug-associated deaths. The number of users of cannabis in 2016 was 192 million worldwide, 34 million for opioids, 34 million for amphetamines, 21 million for ecstasy, and 18 million for cocaine.^2^ For the past several decades, numerous medical complications have been associated with drug abuse. Cocaine has been well known to cause dilated cardiomyopathy, while long-term users of heroin are at greater risk of developing infective endocarditis and cerebral abscesses.^3,4^ Ulcerative cystitis is a known complication associated with the use of ketamine^5^; however, there is emerging evidence of other drugs causing urological effects such as nephrotoxicity, erectile dysfunction (ED), infertility, and obstructive uropathy. Cannabis is found to have some association with bladder and prostate cancer and nonseminomatous germ cell tumors. Heroin and cocaine injections have some association with Fournier’s gangrene. Radiolucent stone formation is associated with cough medicines use. The aim of our review is to have a look into the urological manifestations of the common illicit drugs and their pathophysiology with the clinical presentation.

## Methods

This is a non-systematic critical review of the literature mainly focusing on the use of recreational drugs in urology. Healthcare Databases Advanced Search Export software was used for searching Medline and EMBASE, and also other databases were searched (inception to March 2021). The search was done using MeSH terms: “Cocaine,” “Cannabis,” “Amphetamine,” “Methamphetamine,” “3,4-methylenedioxyN-methylamphetamine,” “NMDA,” “Lysergic Acid Diethylamide,” “Heroin,” “Urological Disease,” “Urologic manifestations,” “Cystitis,” “Neoplasms,” and “Urinary system.” Only articles in English were used. Survey, question, read, recite, and review technique was used for article inclusion.

## Results

### Ketamine

**Background:** In 1970, ketamine was introduced for the first time as a rapid-acting intravenous anesthetic.^6^ Ketamine is lipid-soluble and is rapidly absorbed in the human body through parenteral, nasal, and oral administration routes.^7^ It freely crosses the blood–brain barrier. It produces anesthetic effects by acting as a noncompetitive antagonist of the N-methyl-d-aspartate receptors in the central nervous system.^7,8^

**Clinical Use: **Ketamine is an attractive anesthetic often used in pediatric, obstetric, and hemodynamically unstable patients. Ketamine maintains cardiac output, blood pressure, and heart rate via central sympathetic activation without depressing respiratory function or gut motility.^7^ It is also being used to treat many other health issues like depressive disorders, suicidal ideation, substance use disorders, anxiety disorders, chronic pain, refractory status epilepticus, and bronchial asthma exacerbations.^9^

**Abuse:** Ketamine is known by many “street names,” namely K, jet, super acid, cat Valium, green, and Special K. The two most common administration routes include intranasal (powder snorting and smoking) and oral (tablet and liquid in drinks).^10^ Ketamine abuse is related to so-called dissociative anesthesia. It is characterized by catatonia, catalepsy, and amnesia. In this state, patients become entirely disconnected from their environment. In addition, being short-acting and cheap, it has become a widely used illegal recreational drug among the young population.^11^ In recent studies, it is estimated that 2.3 million young adults used ketamine in the United States in their lifetime.^12^ In Australia, 40% of party drug users reported the use of ketamine, and in 2014, in Hong Kong, more than 2000 cases of ketamine abuse were reported.^12^ Psychological dependence is observed in 79% of abusers after 1 year of regular consumption and withdrawal symptoms after abstinence.^13^

**Urological Implications: **The pathological basis of ketamine-induced urological complications include inflammation, fibrosis, hypersensitivity, microvascular damage, and also direct toxicity. Although the exact mechanism of ketamine-induced urological damage is unclear, however, there is some evidence indicating that ketamine produces metabolites in the urine which induce chemical irritation of the urothelium leading to an inflammatory response in the urothelial tract.^14^ Ketamine causes severe irritation which leads to the transmural inflammation of urothelium, loss of muscle thickness, detrusor muscle fibrosis, and subsequently poor urinary bladder compliance. Research has revealed that ketamine users have raised systemic and local inflammatory markers.^15^ N-methyl-d-aspartate antagonist properties of ketamine may also be affected via a central pathway.^16^

1. **Ketamine and Lower Urinary Tract: **Lower urinary tract symptoms (LUTS) are seen in 50% of ketamine abusers.^17^ Typical presentation includes young patients with mainly storage lower urinary tract symptoms with a background of a history of long-term ketamine abuse. Most common symptoms include increased frequency, urgency, nocturia, small volume voids, and dysuria.^18^
  The LUTS developing in ketamine abusers are attributed to pathological changes taking place in the bladder, commonly referred to as “ketamine cystitis” (KC). Wu et al^19^ proposed 3 clinical stages based on a patient's history of ketamine abuse, laboratory test results, and imaging findings.
  Stage I: Inflammatory stimulation stage (ketamine abuse which lasts less than 2 years; weekly dose of less than 0.5 g, with previous normal renal and liver functions and no bladder and upper tract change).
  Stage II: Initial bladder fibrosis stage (2-4 years of abuse with dose less than 4 g/week, increased gamma-glutamyl transferase (GGT), with bladder wall thickening and decreased bladder capacity).
  Stage III: End stage of bladder fibrosis, contracture stage.
  The evaluation of these patients is variable but usually follows thorough history-taking, clinical examination, and relevant laboratory or other investigations like ultrasound, computed tomography (CT) scan, urodynamics, cystoscopy, etc. In some studies, scoring systems have been used for assessment as well, including International Prostate Symptom Score (IPSS), Interstitial Cystitis Symptom Index (ICSI), and Interstitial Cystitis Problem Index (ICPI).^20^ For KC, abnormal scores include IPSS of more than 8 and the sum of ICSI + ICPI being more than 12 points.
  Bladder imaging often reveals thickening of the bladder wall, reduced bladder capacity, and thickening of the ureteric wall/hydronephrosis in 51% of cases.^21^ Cystoscopic findings include a small capacity bladder with erythematous lesions somewhat similar to carcinoma in situ.^22^
  The treatment part includes a “4-tier” approach as follows:^23^
2. Ketamine cessation.
3. Pharmacological treatment included using nonsteroidal anti-inflammatory drugs (diclofenac and etoricoxib) and anticholinergic agents (solifenacin). In case of partial response, it is followed by opioids (tramadol).
4. Patients not responding to pharmacological treatment are treated like managing interstitial cystitis, using bladder instillation with protective/re-epithelizing agents like sodium hyaluronate and botulinum toxin intravesical injection.
5. Augmentation cystoplasty or cystectomy with urinary diversion may be necessary in severe cases.
6. **Ketamine and Upper Urinary Tract:** Unilateral or bilateral hydronephrosis, often associated with hydroureters, has been found in around 51% of patients having ketamine abuse.^21^ These changes are primarily the result of small bladder capacity in KC as well as the presence of ureteral strictures.^24^ Ketamine-induced bilateral ureteric strictures are often referred to as “walking-stick ureters.”^25^ Ketamine abstinence for more than 1 year has been associated with the resolution of ketamine-induced hydronephrosis.^24^
7. **Ketamine and Urinary Tract Infections: **Thirty percent of patients having KC do suffer from urinary tract infections (UTI). It has been found to be related to the status of upper urinary tract involvement, duration of ketamine use, and severity of LUTS.^26^ In a study, ketamine-associated UTIs were found to be caused by *Escherichia coli* in 50% of the patients and *Enterococcus faecalis* accounted for more than 20%.^26^

### Opioids and Heroin

**Background:** Opioids are derived from opium. The poppy plant (*Papaver somniferum*) is the source of opium. When the petals of the poppy flower wilt completely, its seeds are exposed. The poppy seeds are slit vertically, and opium is extracted and dried. Opioids include naturally present alkaloid morphine or chemically produced derivatives like codeine and heroin. Opioids can bind to mu (µ), delta (δ), and kappa (κ) receptors, which are associated with 3 major classes of endogenous opioid peptides (endorphins, enkephalins, and dynorphins).^27^

**Clinical Use: **Endogenous opioids play a regulatory role in several physiological processes including motor, immunological, gastrointestinal, cardiovascular, neuroendocrine, cognitive, as well as nociceptive functions. Opioid analgesics are used in the treatment of pain associated with musculoskeletal and rheumatological conditions as they have long-lasting effects in reducing pain and are cost-effective.^28^

**Abuse:** Heroin, or morphine diacetate, is a morphine derivative used recreationally. It is most commonly sold as a brown powder that is smoked, snorted, or injected. In the body, it is metabolized into morphine, producing its well-known euphoric and anxiolytic effects. Heroin can rapidly cross the blood–brain barrier once injected and patients usually present with pin-point pupils, depressed mental state, and decreased respiratory rate.^29^ Similarly, methadone and buprenorphine are synthetic or partially synthetic derivatives used in the detoxification process of opioid-dependent patients. They are both used recreationally and consumed in a similar fashion to heroin. 

**Urological Implications:** The urological implications of opioid abuse include effects on testosterone production and on spermatogenesis.

1. **Opioids and Testosterone Production: **Opioids (endogenous as well as exogenous opioids) cause inhibition of hypothalamic gonadotropin-releasing hormone (GnRH) secretion, resulting in disruption of its normal pulsatility and decreased levels of luteinizing hormone. As a consequence, testosterone levels decrease, leading to hypogonadotropic hypogonadism.^27^
  Multiple aspects of sexual dysfunction may consequently arise like decreased libido, ED, and reduced sperm motility.^29^ These symptoms appear to be dose-dependent as higher rates of ED were observed in methadone users due to the higher potency of methadone and its slower rate of renal excretion.^29,30^ Additionally, premature ejaculation has also been noted in opioid abusers, likely as the result of the α-adrenergic blocking effects of opiates.^31^ A study from Taiwan showed a 5-fold higher risk of ED among the heroin-addicted group in comparison to the control group.^32^ A study from Turkey showed that 59.3% of the patients who are opioids users have severe ED and 25.9% have moderate ED according to the International Index of Erectile Function.^33^
2. **Opioids and Spermatogenesis: **Several animal as well as human studies have pointed out that spermatogenesis is affected by opioid use. In a study performed on rats, chronic tramadol use resulted in degenerative changes in the seminiferous tubules, in Sertoli cells, and in Leydig cells.^34^ Another study in rats treated chronically with tramadol found increased caspase-3 and decreased anti-apoptotic protein Bcl-2 expression.^35^ In humans, oligozoospermia has been found in men addicted to opium in comparison to controls.^36^ Furthermore, addicted men had decreased antioxidant activity and a higher DNA fragmentation index.^36^
3. **Opioids and Bladder Cancer:** Numerous case–control studies have linked opioid abuse to an increased risk of bladder cancer.^37,38^ There is a 6-fold increased risk of bladder cancer in heroin users according to a hospital-based case–control studies.^38^ This was attributed to highly mutagenic morphine pyrolysates that may induce sister-chromatid exchanges in cells, directly increasing the risk of developing cancer.^37^ Patients presenting with bladder cancer linked to opioid abuse should present no different from other cases of bladder cancer and should be managed in the same way as non-opioid-induced bladder cancer.
4. **Opioids and Other Urological Issues:** Heroin injection has been associated with cases of acute glans ischemia, penile ulcers, and Fournier’s gangrene. Though the hormonal consequences of heroin abuse subside on opioid-use cessation, opioid-dependent patients are often started on a detoxification program consisting of methadone or buprenorphine to wean their opioid use and manage withdrawal symptoms.^33^ Sexual dysfunction symptoms thus continue to persist due to prolonged, tapering use of opioids. The use of exogenous testosterone has been shown to counter these effects of opioid-induced hypogonadism. Recent studies have demonstrated that synthetic testosterone induces negative feedback on the hypothalamic–pituitary–gonadal axis, which inhibits GnRH and subsequently inhibits spermatogenesis.^39^ Lower rates of sexual dysfunction have been observed in buprenorphine users, possibly due to its reduced effect on testosterone suppression. Patients on methadone maintenance therapy with ED may therefore benefit from a trial of buprenorphine.^39^

### Cocaine

**Background:** Cocaine is extracted from the leaves of the coca plant. It is a sympathomimetic substance.

**Clinical Use:** Its clinical manifestations occur due to α-adrenergic stimulation effects (vasoconstrictive effect) and sympathomimetic effects (due to presynaptic reuptake inhibition of dopamine, norepinephrine, and serotonin).^40^ Before the advent of synthetic derivatives such as lidocaine, cocaine was used medically as a local anesthetic.

**Abuse:** Cocaine is a stimulant and is recreationally sold as a powder that is snorted, smoked, or injected. In patients with cocaine dependence, the signs and symptoms include anxiety, depression, agitation, paranoia, and weakness.^40^

**Urological Implications:** Cocaine affects the urinary and reproductive system in a variety of ways through both local and systemic mechanisms.

1. **Cocaine-Induced Renal Infarction: **Research has shown that cocaine use can cause renal damage; however, renal infarction is a less common entity secondary to cocaine use. Multiple factors are implicated in cocaine-induced renal infarction (CIRI)-like changes in hemodynamics, changes in glomerular matrix synthesis, degradation, and oxidative stress.^41^ However, the exact cause of CIRI is still being elucidated, although some studies have established that it is usually a vascular event secondary to enhanced platelet aggregation, increased thromboxane synthesis, and other vasoconstrictive factors.^42^ Cocaine-induced renal infarction clinically manifests as abdominal/flank pain with onset usually within 2-3 hours after cocaine use. It is often associated with nausea or vomiting and may be associated with elevated temperature.^43^ There is a lack of consensus in relation to the management of CIRI. Treatment options differ widely and patients are managed either conservatively, medically with anticoagulation and aspirin therapy or surgically (nephrectomy).^43^
2. **Cocaine and Priapism:** Cocaine-induced priapism has been associated with intranasal, intraurethral, intracavernosal, and topical use. Cocaine abuse depletes noradrenaline from sympathetic nerve terminals, thereby diminishing the vasoconstriction necessary to remove blood from erectile tissues.^44^ Intracavernosal injection leads to greater production of nitric oxide in the corpus cavernosum, which results in continued erection secondary to continuous Cyclic guanosine monophosphate (cGMP) production.^44^
3. **Cocaine and Fournier’s Gangrene:** Fournier’s gangrene and ischemic necrosis of the penile shaft and scrotum secondary to cocaine injection and topical drug use have been reported in literature. They have been attributed to the potent α-adrenergic vasoconstrictive effects of the drug.^45^

### Amphetamines

**Background**: Amphetamine and its derivatives, methamphetamines and 3,4-methylenedioxy-N-methylamphetamine, are a group of drugs that act on the dopaminergic and serotoninergic systems in the central nervous system, and they are commonly called ecstasy.^46^

**Clinical Use:** Amphetamines are central nervous system stimulants. They are used in the treatment of narcolepsy, obesity, and attention-deficit hyperactivity disorder.^47^

**Abuse: **3,4-Methylenedioxy-N-methylamphetamine is mostly used for recreational purposes. Sold as a white tablet or powder, the drug is usually swallowed or in its powder form smoked or snorted. Ecstasy is used to elicit euphoria, heighten arousal, increase energy, and suppress appetite; following these “highs,” users often experience a period of lethargy known as “coming down.”

## Urological Implications

1. **Amphetamines and Renal Cell Carcinoma: **Amphetamines used as diet pills have been implicated in the development of renal cell carcinoma (RCC). In a study, it has been found to increase the risk of RCC by 2-fold.^48^ There is no explanation for this adverse effect.
2. **Amphetamines and Rhabdomyolysis:** Amphetamines are often taken at clubs and “raves” to increase energy to dance for prolonged periods of time. The following severe strenuous exercise, dehydration, and hyperpyrexia may result in skeletal muscle breakdown and myoglobinuria.^49^ Accumulation of myoglobin in the tubules may subsequently result in acute kidney injury.
3. **Amphetamines and Urinary Retention:** Amphetamines are potent α-adrenergic agonists, and as such, amphetamine-induced stimulation can result in contraction, causing bladder neck closure and dysfunction. Many case reports have reported on this association.^50^
4. **Amphetamines and Sexual Dysfunction:** Male infertility has been associated with amphetamines use. It is understood that drug use influences GnRH secretion, reducing the levels of plasma testosterone in a dose-dependent manner; additionally, sperm DNA damage and tubular degeneration were observed while sperm motility and morphology were unaffected.^51^
  Koro, or genital retraction syndrome, is an unusual delusional disorder associated with increased amphetamine use. Patients present with acute anxiety attacks due to the perceived notion that their penis is shrinking or disappearing.^52^
5. **Amphetamines and Sexually Transmitted Diseases:** Sexually transmitted infections are more common among regular amphetamine users due to an increase in high-risk sexual behavior. Known users should therefore be screened when presenting with common LUTS.

### Cannabis

**Background: **The *Cannabis sativa* plant is the source of cannabis. The biologically active ingredient of cannabis is cannabinoid (tetrahydrocannabinol (THC)). Cannabinoids bind to specific cannabinoid receptors (1 and 2) in the body to produce different clinical manifestations.

**Clinical Use:** Both cannabis and cannabinoids have been used for several medical problems. They have been found useful in the symptomatic treatment of nausea, vomiting, pain, insomnia, anxiety, stress (post-traumatic stress disorder), seizure disorders, as well as Tourette's syndrome.^53^

**Abuse:** The dried leaves and flowering tops of* Cannabis s* *ativa* plants are referred to as marijuana. Marijuana is either smoked or ingested. Stimulation of cannabinoid receptors in the brain produces euphoric or high effects along with an increase in appetite and impaired motor skills and may heighten feelings of anxiety.^54^

## Urological Implications

1. **Cannabis and Urological Cancers:** Cannabis use and bladder cancer have strong associations with an odds ratio of 3.4.^55^ Similarly, marijuana users had an increased risk of prostate cancer (relative risk 3.1; 95% CI: 1.0-9.5).^56^ Also, nonseminomatous germ cell tumors have been found to be strongly associated with cannabis use.^57^ This has been attributed to THC-induced disruption of the hypothalamic–pituitary–testicular axis.
2. **Cannabis and Infertility:** Oligospermia has been found in more than one-third of chronic cannabis smokers, leading to infertility in these patients.^58^ It is again attributed to disruption of the hypothalamic–pituitary–testicular axis.

### Lysergic Acid Diethylamide

**Background: **Lysergic acid diethylamide (LSD) is derived from the ergot fungus. It is a hallucinogenic drug.

**Clinical Use: **It was previously used in the 1950s and 1960s in psychiatric research, which was subsequently halted following its widespread unrestricted use and reports of adverse effects.^59^

**Abuse: **Lysergic acid diethylamide is thought to increase the release of serotonin leading to excitation in the cerebral cortex.^59^ Its use leads to visual hallucinations, terrifying thoughts, as well as, distortions in the perception of time, objects, and color.^60^

## Urological Implications

1. **Retroperitoneal Fibrosis: **Lysergic acid diethylamide abusers may develop retroperitoneal fibrosis. The development of retroperitoneal fibrosis with LSD abuse is thought to be due to LSD’s serotonergic effects. Serotonin has been known to promote the proliferation of myofibroblasts and the deposition of a collagenous matrix to form fibrous tissue.^60,61^ Retroperitoneal fibrosis can lead to obstruction of the ureters. A CT scan can therefore be used to identify the presence of retroperitoneal fibrosis and assess the renal tract for the presence of ureteric obstruction and hydronephrosis. Laboratory tests may also show raised inflammatory markers and deranged renal function.^60^
2. **Genital Mutilation: **Lysergic acid diethylamide abuse has been found to increase the levels of both serotonin and glutamate in the synaptic cleft. Both these neurotransmitters have been associated with the development of hallucinations, which could, in turn, lead to self-mutilation, for example, genital mutilation or self-inflicted testicular amputation; however, the underlying mechanism is still unclear.^61^

## Discussion

The recently released United Nations World Drug report has provided estimates of and trends in drug use. As per the information provided, there is an increase in the population at risk of drug abuse by 2030, with 43% and 10% being in low- and middle-income countries, respectively. This is going to be a global challenge as around 1 billion and 6 billion people live in low- and middle-income countries. Drug abuse impacts individuals individually (both physically and mentally) as well as socially, and they often need medical care for various health-related issues arising directly or indirectly due to drug abuse. This makes it imperative for healthcare professionals to be well aware of different manifestations of substance abuse.

Illicit drug abuse can have an adverse impact on the urological system and can present with unknown and unexpected clinical manifestations leading to referrals to urologists. This review highlights and summarizes different clinical manifestations as well as pathological impact of recreational and illicit drug abuse on the urological system (Table 1). The presentations can be varied and often challenging for urologists due to atypical presentations making it necessary for urologists to be aware of the myriad of genitourinary complications. The “CHECKS” acronym is a useful tool to help in urological diagnosis when aetiology is not clinically obvious and illicit drug abuse is suspected. This acronym was introduced by Coull and O’Brien^62^ and stands for cocaine, heroin, ecstasy, cannabis, ketamine, and steroids.

## Conclusion

Illicit drug abuse among youth is widespread and is growing with every passing day. These may have adverse effects on different systems including the urological organ system. We strongly recommend that thorough history needs to be elicited particularly if young patients present with LUTS, sexual dysfunction, infertility, or any other atypical presentation. Further studies are required to analyze pathophysiology and to determine appropriate testing and treatment.

## Figures and Tables

**Table 1.** Summarized Results of Urological Complications Due to Illicit Drug

**Part Affected**	**Complication**	**Illicit Drug**
Upper tract	Renal cell carcinoma	Amphetamine
	Renal infarction	Cocaine
	Ureteric obstruction	Ketamine
	Retroperitoneal fibrosis	Lysergic acid diethylamide
Rhabdomyolysis	Amphetamine
Lower tract	Bladder cancer	Cannabis, heroin
	Prostate cancer	Cannabis
	Cystitis and LUTS	Ketamine
Urinary obstruction	Amphetamine
Male genitalia	Testicular cancer	Cannabis
	Genital self-mutilation	Lysergic acid diethylamide
	Testicular self-mutilation	Lysergic acid diethylamide
	Fournier’s gangrene	Cocaine
Glans Ischemia	Heroin
Sexual dysfunction	Erectile dysfunction	Amphetamine, cannabis, heroin
	Priapism	Cocaine
	Oligospermia/infertility	Cannabis, amphetamine, heroin
	Testosterone insufficiency	Heroin
Sexually transmitted disease	Amphetamine

LUTS, lower urinary tract symptoms.

## References

[1] United Nations Office on Drugs and Crime UNODC, World Drug Report 2018 Analysis of drug markets. ISBN: 978-92-1-148304-8; eISBN: 978-92-1-045058-4; United Nations publication, Sales No. E.18.XI.9. 2018.

[2] Smith KB Smith CA . The relational approach to presidential communication. The ford/carter coalitions. Journal of Applied Communication Research. 1979;7(2):135 152. 10.1080/00909887909365203)

[3] Enevoldson TP Recreational drugs and their neurological. Neurol Pract. 2004;75:3. 10.1136/jnnp.2004.045732 PMC1765663 15316039

[4] Robledo-Carmona J Ortega-Jimenez MV García-Pinilla JM Cabra B de Teresa E . Severe cardiomyopathy associated to cocaine abuse. Int J Cardiol. 2006;112(1):130 131. 10.1016/j.ijcard.2005.09.058) 16376443

[5] Winstock AR Mitcheson L Gillatt DA Cottrell AM . The prevalence and natural history of urinary symptoms among recreational ketamine users. BJU Int. 2012;110(11):1762 1766. 10.1111/j.1464-410X.2012.11028.x) 22416998

[6] Peltoniemi MA Hagelberg NM Olkkola KT Saari TI . Ketamine:a review of clinical pharmacokinetics and pharmacodynamics in anesthesia and pain therapy. Clin Pharmacokinet. 2016;55(9):1059 1077. 10.1007/s40262-016-0383-6) 27028535

[7] Zanos P Moaddel R Morris PJ et al. Ketamine and ketamine metabolite pharmacology: insights into therapeutic mechanisms. Pharmacol Rev. 2018;70(3):621 660. 10.1124/pr.117.015198) 29945898 PMC6020109

[8] Myers FA Bluth MH Cheung WW . Ketamine: a cause of urinary tract dysfunction. Clin Lab Med. 2016;36(4):721 744. 10.1016/j.cll.2016.07.008) 27842789

[9] Radvansky BM Puri S Sifonios AN Eloy JD Le V . Ketamine-A narrative review of its uses in medicine. Am J Ther. 2016 23(6):e1414-e1426. 10.1097/MJT.0000000000000257) 25923225

[10] Drug Enforcement Administration. Ketamine. Available at: https://www. dea.gov/factsheets/ketamine. Accessed December 30, 2019.

[11] Bokor G Anderson PD . Ketamine: an update on its abuse. J Pharm Pract. 2014;27(6):582 586. 10.1177/0897190014525754) 24651639

[12] Liu Y Lin D Wu B Zhou W . Ketamine abuse potential and use disorder. Brain Res Bull. 2016;126(1):68 73. 10.1016/j.brainresbull.2016.05.016) 27261367

[13] Chen R Lee A Chan R . Report on A Study on the Cognitive Impairment and Other Harmful Effects Caused by Ketamine Abuse. A study on the cognitive impairment and other harmful effects from ecstasy and ketamine abuse. Hong Kong , Chinese: Narcotics Division, Security Bureau. The Government of the Hong Kong Special Administrative Region; 2004.

[14] Chu PS Ng CF Ma WK . Ketamine uropathy: Hong Kong experience. In: Yew DT ed. Ketamine: Use and Abuse. CRC Press; Boca Raton; 2015:207 226.

[15] Division of Urology, Department of Surgery, Princess Margaret Hospital, Tuen Mun Hospital. Research on urological sequelae of ketamine abuse. Available at: http://www.nd.gov.hk/pdf/20110307_beat_drug_fund_ report.pdf.

[16] Jankovic SM Jankovic SV Stojadinovic D Jakovljevic M Milovanovic D . Effect of exogenous glutamate and N-methyl-D-aspartic acid on spontaneous activity of isolated human ureter. Int J Urol. 2007;14(9):833 837. 10.1111/j.1442-2042.2007.01834.x) 17760751

[17] Li CC Wu ST Cha TL Sun GH Yu DS Meng E . A survey for ketamine abuse and its relation to the lower urinary tract symptoms in Taiwan [sci rep.]. Sci Rep. 2019;9(1):7240. 10.1038/s41598-019-43746-x) 31076629 PMC6510790

[18] Chen CH Lee MH Chen YC Lin MF . Ketamine-snorting associated cystitis. J Formos Med Assoc. 2011;110(12):787 791. 10.1016/j.jfma.2011.11.010) 22248834

[19] Wu P Wang Q Huang Z Wang J Wu Q Lin T . Clinical staging of ketamine-associated urinary dysfunction: a strategy for assessment and treatment. World J Urol. 2016;34(9):1329 1336. 10.1007/s00345-016-1759-9) 26803767

[20] Yang SS Jang MY Lee KH et al. Sexual and bladder dysfunction in male ketamine abusers: a large-scale questionnaire study. PLOS ONE. 2018;13(11):e0207927. 10.1371/journal.pone.0207927) PMC6261603 30485367

[21] Chu PS-K Ma WK Wong SC et al. The destruction of the lower urinary tract by ketamine abuse: a new syndrome? BJU Int. 2008;102(11):1616 1622. 10.1111/j.1464-410X.2008.07920.x) 18680495

[22] Oxley JD Cottrell AM Adams S Gillatt D . Ketamine cystitis as a mimic of carcinoma in situ. Histopathology. 2009;55(6):705 708. 10.1111/j.1365-2559.2009.03437.x) 19919587

[23] Castellani D Pirola GM Gubbiotti M et al. What urologists need to know about ketamine-induced uropathy: a systematic review. Neurourol Urodyn. 2020;39(4):1049 1062. 10.1002/nau.24341) 32212278

[24] Yee CH Teoh JY Lai PT et al. The risk of upper urinary tract involvement in patients With ketamine-associated uropathy. Int Neurourol J. 2017;21(2):128 132. 10.5213/inj.1732704.352) 28673061 PMC5497195

[25] Huang PW Meng E Cha TL Sun GH Yu DS Chang SY . ‘Walking-stick ureters’ in ketamine abuse. Kidney Int. 2011;80(8):895. 10.1038/ki.2011.242) 21960173

[26] Liu W Wu W Wei Y et al. Epidemiologic characteristics and risk factors in patients with ketamine-associated lower urinary tract symptoms accompanied by urinary tract infection: A cross-sectional study. Medicine. 2019;98(23):e15943. 10.1097/MD.0000000000015943) PMC6571413 31169717

[27] Vuong C Van Uum SH O'Dell LE Lutfy K Friedman TC . The effects of opioids and opioid analogs on animal and human endocrine systems. Endocr Rev. 2010;31(1):98 132. 10.1210/er.2009-0009) 19903933 PMC2852206

[28] Kosten TR O'Connor PG . Management of drug and alcohol withdrawal. N Engl J Med. 2003;348(18):1786 1795. 10.1056/NEJMra020617) 12724485

[29] Kulkarni M Hayden C Kayes O . Recreational drugs and male fertility. Trends Urology & Men Health. 2014;5(5):19 23. 10.1002/tre.414)

[30] Srivastava A Kahan M Nader M . Primary care management of opioid use disorders: abstinence, methadone, or buprenorphine-naloxone? Can Fam Physician. 2017;63(3):200 205. 28292795 PMC5349718

[31] Bonanome A Grundy SM . Effect of dietary stearic acid on plasma cholesterol and lipoprotein levels. N Engl J Med. 1988;318(19):1244 1248. 10.1056/NEJM198805123181905) 3362176

[32] Bang-Ping J Sexual dysfunction in men who abuse illicit drugs: A preliminary report. J Sex Med. 2009;6(4):1072 1080. 10.1111/j.1743-6109.2007.00707.x) 18093094

[33] Kumsar NA Kumsar Ş Dilbaz N . Sexual dysfunction in men diagnosed as substance use disorder. Andrologia. 2016;48(10):1229 1235. 10.1111/and.12566) 26940022

[34] Abdellatief RB Elgamal DA Mohamed EE . Effects of chronic tramadol administration on testicular tissue in rats: an experimental study. Andrologia. 2015;47(6):674 679. 10.1111/and.12316) 25228095

[35] Ibrahim MA Salah-Eldin AE . Chronic addiction to tramadol and withdrawal effect on the spermatogenesis and testicular tissues in adult male albino rats. Pharmacology. 2019;103(3-4):202 211. 10.1159/000496424) 30699432

[36] Safarinejad MR Asgari SA Farshi A et al. The effects of opiate consumption on serum reproductive hormone levels, sperm parameters, seminal plasma antioxidant capacity and sperm DNA integrity. Reprod Toxicol. 2013;36:18 23. 10.1016/j.reprotox.2012.11.010) 23207164

[37] Cioe PA Friedmann PD Stein MD . Erectile dysfunction in opioid users: lack of association with serum testosterone. J Addict Dis. 2010;29(4):455 460. 10.1080/10550887.2010.509279) 20924881 PMC2951625

[38] Hosseini SY Safarinejad MR Amini E Hooshyar H . Opium consumption and risk of bladder cancer: a case-control analysis. Urol Oncol. 2010;28(6):610 616. 10.1016/j.urolonc.2008.10.016) 19110453

[39] Abs R Verhelst J Maeyaert J et al. Endocrine consequences of long-term intrathecal administration of opioids. J Clin Endocrinol Metab. 2014;85:2215 2222. 10.1210/jcem.85.6.6615 10852454

[40] Warner EA Cocaine abuse. Ann Intern Med. 1993;119(3):226 235. 10.7326/0003-4819-119-3-199308010-00009) 8323092

[41] Dressler FA Malekzadeh S Roberts WC . Quantitative analysis of amounts of coronary arterial narrowing in cocaine addicts. Am J Cardiol. 1990;65(5):303 308. 10.1016/0002-9149(90)90292-9) 2301258

[42] Kohan DE Endothelins in the normal and diseased kidney. Am J Kidney Dis. 1997;29(1):2 26. 10.1016/s0272-6386(97)90004-4) 9002526

[43] Bemanian S Motallebi M Nosrati SM . Cocaine-induced renal infarction: report of a case and review of the literature. BMC Nephrol. 2005;6:10. 10.1186/1471-2369-6-10) PMC1253515 16176587

[44] Skeldon SC Goldenberg SL . Urological complications of illicit drug use. Nat Rev Urol. 2014;11(3):169 177. 10.1038/nrurol.2014.22) 24535583

[45] Khan F Mukhtar S Anjum F et al. Fournier's gangrene associated with intradermal injection of cocaine. J Sex Med. 2013;10(4):1184 1186. 10.1111/jsm.12055) 23347293

[46] Kalant H The pharmacology and toxicology of ‘ecstasy’ (MDMA) and related drugs. CMAJ. 2001;165(7):917 928. 11599334 PMC81503

[47] Heal DJ Smith SL Gosden J Nutt DJ . Amphetamine, past and present--a pharmacological and clinical perspective. J Psychopharmacol. 2013;27(6):479 496. 10.1177/0269881113482532) 23539642 PMC3666194

[48] Dunnick JK Eustis SL . Decreases in spontaneous tumors in rats and mice after treatment with amphetamine. Toxicology. 1991;67(3):325 332. 10.1016/0300-483x(91)90031-u) 2048132

[49] Richards JR Johnson EB Stark RW Derlet RW . Methamphetamine abuse and rhabdomyolysis in the ED: a 5-year study. Am J Emerg Med. 1999;17(7):681 685. 10.1016/s0735-6757(99)90159-6) 10597089

[50] Beuerle JR Barrueto F . Neurogenic bladder and chronic urinary retention associated with MDMA abuse. J Med Toxicol. 2008;4(2):106 108. 10.1007/BF03160964) 18570171 PMC3550134

[51] Fronczak CM Kim ED Barqawi AB . The insults of illicit drug use on male fertility. J Androl. 2012;33(4):515 528. 10.2164/jandrol.110.011874) 21799144

[52] Garlipp P Koro - A culture-bound phenomenon intercultural psychiatric implications. Ger J Psychiatry. 2008;11:21 28.

[53] Klumpers LE Thacker DL . A brief background on cannabis: from plant to medical indications. J AOAC Int. 2019;102(2):412 420. 10.5740/jaoacint.18-0208) 30139415

[54] Vescovi PP Pedrazzoni M Michelini M Maninetti L Bernardelli F Passeri M . Chronic effects of marihuana smoking on luteinizing hormone, follicle-stimulating hormone and prolactin levels in human males. Drug Alcohol Depend. 1992;30(1):59 63. 10.1016/0376-8716(92)90036-c) 1591981

[55] Chacko JA Heiner JG Siu W Macy M Terris MK . Association between marijuana use and transitional cell carcinoma. Urology. 2006;67(1):100 104. 10.1016/j.urology.2005.07.005) 16413342

[56] Sidney S Quesenberry CP Friedman GD Tekawa IS . Marijuana use and cancer incidence (California, United States). Cancer Causes Control. 1997;8(5):722 728. 10.1023/a:1018427320658) 9328194

[57] Daling JR Doody DR Sun X et al. Association of marijuana use and the incidence of testicular germ cell tumors. Cancer. 2009;115(6):1215 1223. 10.1002/cncr.24159) 19204904 PMC2759698

[58] Kolodny RC Masters WH Kolodner RM Toro G . Depression of plasma testosterone levels after chronic intensive marihuana use. N Engl J Med. 1974;290(16):872 874. 10.1056/NEJM197404182901602) 4816961

[59] Liechti ME Modern clinical research on LSD. Neuropsychopharmacology. 2017;42(11):2114 2127. 10.1038/npp.2017.86) 28447622 PMC5603820

[60] Liester MB A review of lysergic acid diethylamide (LSD) in the treatment of addictions: historical perspectives and future prospects. Curr Drug Abuse Rev. 2014;7(3):146 156. 10.2174/1874473708666150107120522) 25563445

[61] Sotiropoulos A Injury to the bladder. Unusual Complication of Lysergie Acid diethylamide. Urology. 1974;3(6):755 758. 10.1016/s0090-4295(74)80219-0) 4836338

[62] Coull N O’Brien T . ‘Street urology’: beyond the formulary. BJU Int. 2009;103(6):721 722. 10.1111/j.1464-410X.2008.08077.x) 18990155

